# Regional gray matter volumetric changes in autism associated with social and repetitive behavior symptoms

**DOI:** 10.1186/1471-244X-6-56

**Published:** 2006-12-13

**Authors:** Donald C Rojas, Eric Peterson, Erin Winterrowd, Martin L Reite, Sally J Rogers, Jason R Tregellas

**Affiliations:** 1 Department of Psychiatry, University of Colorado Health Sciences Center, Denver, CO, 80220, USA; 2 Department of Psychology, Colorado State University, Fort Collins, CO, 80523, USA; 3 Department of Psychiatry and M.I.N.D. Institute, University of California at Davis, Sacramento, CA, 95817, USA

## Abstract

**Background:**

Although differences in brain anatomy in autism have been difficult to replicate using manual tracing methods, automated whole brain analyses have begun to find consistent differences in regions of the brain associated with the social cognitive processes that are often impaired in autism. We attempted to replicate these whole brain studies and to correlate regional volume changes with several autism symptom measures.

**Methods:**

We performed MRI scans on 24 individuals diagnosed with DSM-IV autistic disorder and compared those to scans from 23 healthy comparison subjects matched on age. All participants were male. Whole brain, voxel-wise analyses of regional gray matter volume were conducted using voxel-based morphometry (VBM).

**Results:**

Controlling for age and total gray matter volume, the volumes of the medial frontal gyri, left pre-central gyrus, right post-central gyrus, right fusiform gyrus, caudate nuclei and the left hippocampus were larger in the autism group relative to controls. Regions exhibiting smaller volumes in the autism group were observed exclusively in the cerebellum. Significant partial correlations were found between the volumes of the caudate nuclei, multiple frontal and temporal regions, the cerebellum and a measure of repetitive behaviors, controlling for total gray matter volume. Social and communication deficits in autism were also associated with caudate, cerebellar, and precuneus volumes, as well as with frontal and temporal lobe regional volumes.

**Conclusion:**

Gray matter enlargement was observed in areas that have been functionally identified as important in social-cognitive processes, such as the medial frontal gyri, sensorimotor cortex and middle temporal gyrus. Additionally, we have shown that VBM is sensitive to associations between social and repetitive behaviors and regional brain volumes in autism.

## Background

Studies of volumetric quantification of magnetic resonance imaging (MRI) data in autism have largely been non-replicative. To illustrate with a single example of considerable theoretical importance to autism, interest in hippocampal volume has been high since the neuropathology studies of Bauman and Kemper [1] reported evidence of increased cell packing density and reduced cell size in the hippocampus. However, as pointed out in two recent articles [2,3], there is little agreement between studies measuring hippocampal volume in individuals with autism. To date, there are 4 studies reporting no differences [4-7], 3 reports of decreased volume in autism relative to controls [8,9], and 3 reports of increased hippocampal volume in autism [2,3,10]. The differences among the studies are considerable, including different sample characteristics, MRI scanners, methods for assessing hippocampal volume and statistical approaches for analyzing data (reviewed in part in Rojas et al. [2]; see also [11]), and no single, clear candidate emerges to account for the varying findings for the hippocampus.

There are a variety of potentially confounding factors in autism research that challenge the scientific community that are not MRI specific, such as age, gender, IQ, and the inherent heterogeneity of the disorder separate from these other factors (reviewed in Palmen and van Engeland [12]). For example, Palmen and van Engeland [12] accept the argument made by Piven and Arndt [13] that IQ must be strictly matched or statistically factored out in autism studies, and their conclusions on cerebellar studies in autism are based on dismissal of the various studies that do not adhere to this recommendation (e.g., [14-17]). In contrast, Yeung-Courchesne and Courchesne [18] argued strongly that in attempting to "control" for a domain non-specific construct such as IQ, variability truly associated with autism could be discarded as "non-specific". Indeed, few MRI studies critically examine the effects of using IQ matched, but etiologically heterogeneous samples of developmentally disordered controls. The alternate use of statistical covariates for IQ is also usually performed without consideration for the assumptions underlying such analyses (e.g., correlation between the covariate and dependent measure in the sample being measured).

In commenting on differences in major findings between MRI studies, methodological differences are often appealing candidates. Differences in MRI scanner hardware and acquisition hardware can lead to differences across sites (e.g., see Patwardhan et al. [19]) that are difficult to assess. Compounding this problem, many, if not most of the MRI-based morphometric literature in autism have employed manual tracing methods. Manual tracing methods suffer from several problems that negatively impact their reliability and validity. First, they rely on sulcal/gyral boundary determinations that are relatively arbitrarily determined and are highly variable from individual brain to brain. Second, those sulcal/gyral boundaries are often not well correlated with histoanatomically defined areas [e.g., [20-22]]. Third, they are susceptible to rater judgments, which necessarily introduce inter-rater, and therefore intra- and inter-laboratory error variance into the comparisons. Individual laboratories develop highly reliable protocols for such boundary determination, but these protocols are not standardized and are challenging to adapt between sites.

Despite the difficulties inherent with manually traced structural MRI studies, a small number of findings appear to replicate across laboratories. The most consistent finding has been that brain volume appears to be larger in autism [23-26], although there is emerging consensus that this finding is restricted to young children with autism [27,28]. Differences in cerebellar volume are reported in many studies [15-17,29-34], but the directionality of the finding in autism (larger or smaller relative to comparison group) and region within the cerebellum (vermis or hemisphere) differ greatly between studies. Most studies of the cerebellum, however, report reduced vermal volume in autism, unless IQ is matched or covaried in the analysis (e.g., see [12] for a review). Some studies have reported enlarged caudate nuclei [35-37], although this finding does not always survive correction for overall brain volume [e.g., [35]] and another study failed to observe enlargement with or without correction for total brain volume [38]. Enlargement of the caudate nucleus has been correlated with repetitive/stereotyped behaviors in two studies [35,36].

Several automated approaches to MRI morphometry have been developed over the past decade, including template-based, region of interest (ROI) labeling procedures (e.g., see [39-42]), cortical thickness approaches [43,44] and voxel based morphometry (VBM [45-47]). Some of these approaches have been applied to studies of autism spectrum disorders [44,48-55], particularly using VBM methods. In the first VBM study of 15 people with autism, Abell et al. [48] identified abnormalities in the frontal cortex, parahippocampal gyrus, fusiform gyrus, occipital-temporal junction, and the cerebellum [48]. Waiter et al. [54] obtained significant findings in similar regions, although the directionality of the findings (increase or decrease) for some structures (e.g., left inferior frontal gyrus) was opposite to Abell et al. [48]. McAlonen et al. [51] identified reductions in fronto-striatal regions. Two additional VBM studies have limited their analyses to regions associated with social cognition. Boddaert et al. [49] reported bilateral gray matter (GM) decreases in the superior temporal sulcus, a region whose activity has been reported to be linked with biological motion perception and theory of mind [56]. Salmond et al. [55] identified abnormalities in the amygdala and hippocampal region, the orbital frontal cortex, the superior temporal gyri and the cerebellum. Using a related technique, but focusing on GM thickness rather than volume, Hadjikhani et al. [57] found GM thinning in autism in regions associated with the so-called mirror-neuron system, including pre- and post-central gyri, inferior frontal gyrus, medial frontal gyrus and middle temporal gyrus, all of which were significantly correlated with social and communication deficits in the autism participants. In summary, studies using automated volumetric and/or thickness measurements suggest that autism may be associated with GM differences in distributed cortical and subcortical regions that are important in social cognitive and/or motor processes.

VBM studies in autism have not, however, capitalized on the ability of the method to do whole-brain correlations between GM and symptom measures. In the current study, we applied VBM methods to a well defined sample of patients with autistic disorder with the goal of replicating prior whole brain findings in the literature reported for both gray and white matter. In addition, we examined correlations between GM volume, IQ and autism symptom measures derived from diagnostic interviews. We hypothesized that caudate nucleus volume would correlate with repetitive behaviors in autism, replicating previous hand tracing results. Finally, we hypothesized that social communication deficits would correlate with volumes of structures known to participate in social cognitive processes such as the amygdala, medial frontal gyri and superior temporal sulcus.

## Methods

### Participants and behavioral measures

Twenty-four males with autism between the ages of 7 and 47 years (mean: 22.60 ± 11.61 years) were recruited to participate in the study. Individuals with autism were included in that group if they met clinical criteria for DSM-IV autistic disorder [58], as well as criteria for autism on both the Autism Diagnostic Interview (ADI: Lord et al. [59]) and the Autism Diagnostic Observation Schedule (ADOS: Lord et al. [60]). The autism participants were tested, either by karyotype or DNA testing, for fragile X syndrome, and were all negative. For comparison, 23 male participants with no history of neurological or psychiatric disorders were recruited from the Denver metropolitan region. Comparison subjects were first screened using the Structured Clinical Interview, DSM-IV (SCID) Screen Patient Questionnaire – Extended [61], and subjects whose responses needed further clarification were administered that portion of the full SCID interview [62]still in question. Participants in the comparison group had no personal history of neurological or Axis I psychiatric illness and met Research Diagnostic Criteria for never mentally-ill [63]. In addition, comparison subjects had no reported family history of neurological or psychiatric illness among first-degree relatives.

All participants were administered either a full Wechsler Adult Intelligence Scale, 3^rd ^edition (WAIS-III; [64]) or Wechsler Intelligence Scale for Children, 3^rd ^edition (WISC-III; [65]). Demographic and cognitive data on participants are shown in Table 1. All subjects signed informed consent to participate in the experiment consistent with the guidelines of the Colorado Multiple Institution Review Board.

**Table 1:** Demographic characteristics and whole brain volume measures for the study groups (mean ± SD).

	AD (N = 24)		Control (N = 23)	
	Mean (SD)	Range	Mean (SD)	Range

Age (y)	20.79 (10.58)	7.8–44	21.41 (10.91)	7.8–44
Education (y)	10.42 (3.43)	6–16	12.09 (5.06)	6–20
VIQ*	94.96 (20.98)	55–134	118.00 (13.18)	92–134
PIQ*	95.67 (18.84)	62–128	115.73 (9.57)	100–138
FSIQ*	94.75 (20.64)	60–133	118.74 (11.18)	99–139
Total Gray Matter (ml)	758.92 (62.63)	650–895	771.95 (60.28)	617–901
Total White Matter (ml)	444.10 (50.83)	357–553	463.32 (44.99)	359–570
Brain/Intracranial ratio	.68 (.03)	.61–.72	.67 (.04)	.57–.71

VIQ = Verbal intelligence quotient (IQ), PIQ = Performance IQ, FSIQ = Full scale IQ; * p < .05;

### Image acquisition

Spoiled gradient recalled (SPGR) T1-weighted (TR = 40 ms, TE = 5 ms, 40 degree flip angle, NEX = 1) images were acquired on a 1.5 T General Electric Signa system (G.E. Medical Systems, Inc., Milwaukee, WI). One-hundred and twenty-four contiguous, 1.7-mm thick coronal slices with a 192 × 256 (reconstructed to 256^2^) matrix in a 240 mm field of view resulted in voxel dimensions of .9375 by .9375 by 1.7 mm. Prior to the coronal scan sequence, the coronal slice axis was aligned perpendicular to a straight line connecting the anterior tip of the genu of the corpus callosum with the posterior tip of the splenium of the corpus callosum in a sagittal scout image series. Participants were not sedated for the scans.

### Image processing

Image processing and statistical analyses were conducted using the SPM2 software package [66]. To account for potential differences between our local sample and the templates provided in SPM2, an "optimized VBM" protocol was used, as described in Good et al. [67], which results in the use of a study-specific anatomical template for spatial normalization and tissue segmentation, as described below.

To create the customized template, all scans were first segmented into gray, white and cerebrospinal fluid (CSF) compartments in their native space using the standard SPM2 T1 template (MNI305 [68]). The spatial normalization parameters for individual subjects were then estimated based on the individual GM compartments (7 × 9 × 7 discrete cosine basis functions, 16 non-linear iterations, .01 regularization) and then applied to the original native T1 data. These normalized images were segmented again using the MNI305 template and spatial priors, and averages of the 51 T1 images, as well as corresponding GM, white matter and CSF averages from the segmentation output. These four averages were then smoothed with an isotropic 8 mm FWHM Gaussian kernel to produce a new study specific template, as well as spatial priors for gray, white and CSF compartments.

The original, native T1 images were then segmented using the new template and priors, and the spatial normalization parameters were estimated using the GM compartment and applied to the native images to produce new spatially normalized images. These were segmented again in normalized space and multiplied (i.e., modulated) by the Jacobian determinants from the normalization step to preserve the volume information in each voxel [67]. The modulated GM images were then smoothed with a 12 mm FWHM Gaussian kernel for statistical analyses.

### Statistical analyses

To examine differences in regional GM volume between groups, an ANCOVA model was employed with two covariates, total GM volume and age. Total GM volume was employed so that inferences could be made about regional differences in volume. Age was employed as a covariate in consideration of both the wide age range of the sample, the possible age-dependency of structural findings in autism [e.g., [27]], and preliminary examination of age correlations with the hypothesized regions of interest (see below). Based on the results of the age correlation analysis (see below), the linear effect of age was entered as a covariate to account for the confounding effects of age on regional GM volume.

Based on previous findings in autism, we decided on a list of structures to use as a priori regions of interest (ROI) for our analyses to increase the sensitivity of our group comparisons. For each ROI, if a cluster of 25 or more contiguous voxels exceeded an uncorrected p < .01 threshold at the local maximum voxel, we applied a small volume correction (SVC: Worseley et al. [69]) at p < .05 on a 6 mm diameter sphere centered on the cluster maximum. Non-hypothesized group differences (i.e., outside of regions of interest) were only considered if they reached significance after correcting for proportion of false positive results using a false discovery rate (FDR) threshold of p < .05. Whether a cluster was considered part of an ROI or not was determined from whether the local maximum voxel was contained within the label of the same name in the Automated Anatomical Labeling (AAL) atlas [70]. Our hypothesized bilateral ROIs, based on regions associated with motor behavior and social cognition, were: 1) medial frontal gyrus, 2) inferior frontal gyrus, 3) middle and superior temporal gyrus (thus containing superior temporal sulcus), 4) fusiform gyrus, 5) cerebellum, 6) posterior cingulate gyrus (i.e., precuneus/retrosplenial cortex), 7) pre-central gyrus, 8) post-central gyrus, 9) amygdala, 10) caudate nucleus and 11) hippocampus. The hippocampus was included because there is significant overlap (N = 15 with autism) between this sample and that reported by Rojas et al. [2], who reported significantly larger hippocampi in adults with autism.

For partial correlations with symptom domains, a multiple regression analysis with the ADI-R, Social and Communication Total score, ADI-R Repetitive and Stereotyped Patterns score and a measure of severity for the ADOS, along with total gray matter volume, was computed in SPM2. The ADOS severity measure [71] was calculated by summing the Social and Communication scores and dividing by the number of items from which the total is derived (i.e., to account for differences in the ADOS modules, which differ by developmental level). Symptom correlation analyses were restricted to regions contained within a mask including the structures listed for the group comparison above and were evaluated for significance using the small volume correction (p < .05). Age and FSIQ were also evaluated for significance using the ROI mask, but because there were no a priori hypotheses, we used a more conservative multiple comparison correction of FDR < .05.

## Results

### Sample characteristics

Although the autism group had significantly lower full scale (t(45) = 4.92, p < .05), verbal (t(45) = 4.48, p < .05) and performance (t(45) = 4.57, p < .05) IQ scores than the control group, no differences in age, education or handedness were observed between groups (all p > .10, see Table 1). Verbal, Performance, and Full scale IQ, however, were not significantly correlated with the volumes of any of the voxels within the structural ROIs in the study, even at an uncorrected p < .01, in partial correlation analyses controlling for total GM volume.

**Figure 1:**
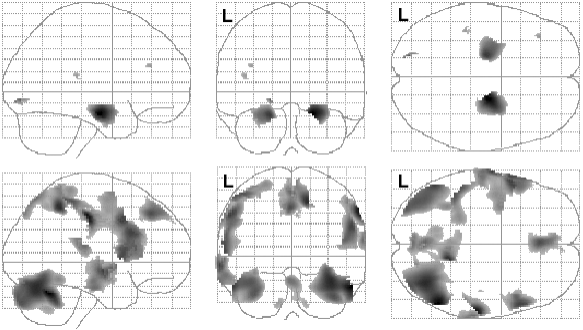
Correlations between regional gray matter volume and age. Positive (top row) and negative (bottom row) partial correlations, corrected for total gray matter volume, are shown in glass brain projection. SPM(*t*) map display threshold is FDR < .05. L = left hemisphere. The data in this figure correspond to the linear effects reported in Table 2.

A preliminary voxel-wise partial correlation analysis of age (with linear, quadratic and cubic polynomial age expansions to account for linear and non-linear age effects) controlling for total GM volume, revealed that significant correlations existed with some of our structures of interest (Figure 1). There were significant linear effects, but no significant quadratic or cubic effects (Table 2).

**Table 2:** Significant partial correlations between regional GM and age, controlling for total gray matter volume

**REGIONS**	**T**	**CLUSTER SIZE**	**MNI COORDINATES**		
Left Hippocampus*	5.87	3957	-20	-8	-17
Right Hippocampus*	7.02	4088	23	-13	-19
Left Lingual Gyrus	5.38	102	-19	-84	-6
Left Superior Temporal Gyrus	3.91	76	-41	-32	16
Left Cerebellar Crus I	-4.75	12797	-54	-60	-27
Right Cerebellar Crus I	-5.85	23078	55	-58	-28
Right Supramarginal Gyrus	-5.66	1774	61	-23	43
Right Inferior Frontal Gyrus, Pars Opercularis	-4.65	5987	55	18	27
Right Precuneus	-5.07	7418	15	-47	50
Left Precentral Gyrus	-4.80	12454	-58	8	38
Left Middle Temporal Gyrus	-4.77	4005	-66	-8	-8

Notes: The sign of the t-statistic indicates whether the correlation coefficients are positive or negative partial correlations. Regions reported in table are significant at FDR < .05. ^† ^All labels are derived from AAL atlas (see text). * The super-threshold voxels in these two clusters overlap the majority of the amygdala both left and right hemispheres, as well as the hippocampus. Only linear effects are shown because no quadratic or cubic effects remained after correcting for multiple comparisons.

### Total gray matter, white matter, and brain/intracranial ratios

No differences were observed between groups for total GM, total white matter or brain (gray + white) to intracranial (gray + white + csf) volume ratio, regardless of whether age was used as a covariate or not (Table 1).

### Regional gray matter results

**Figure 2:**
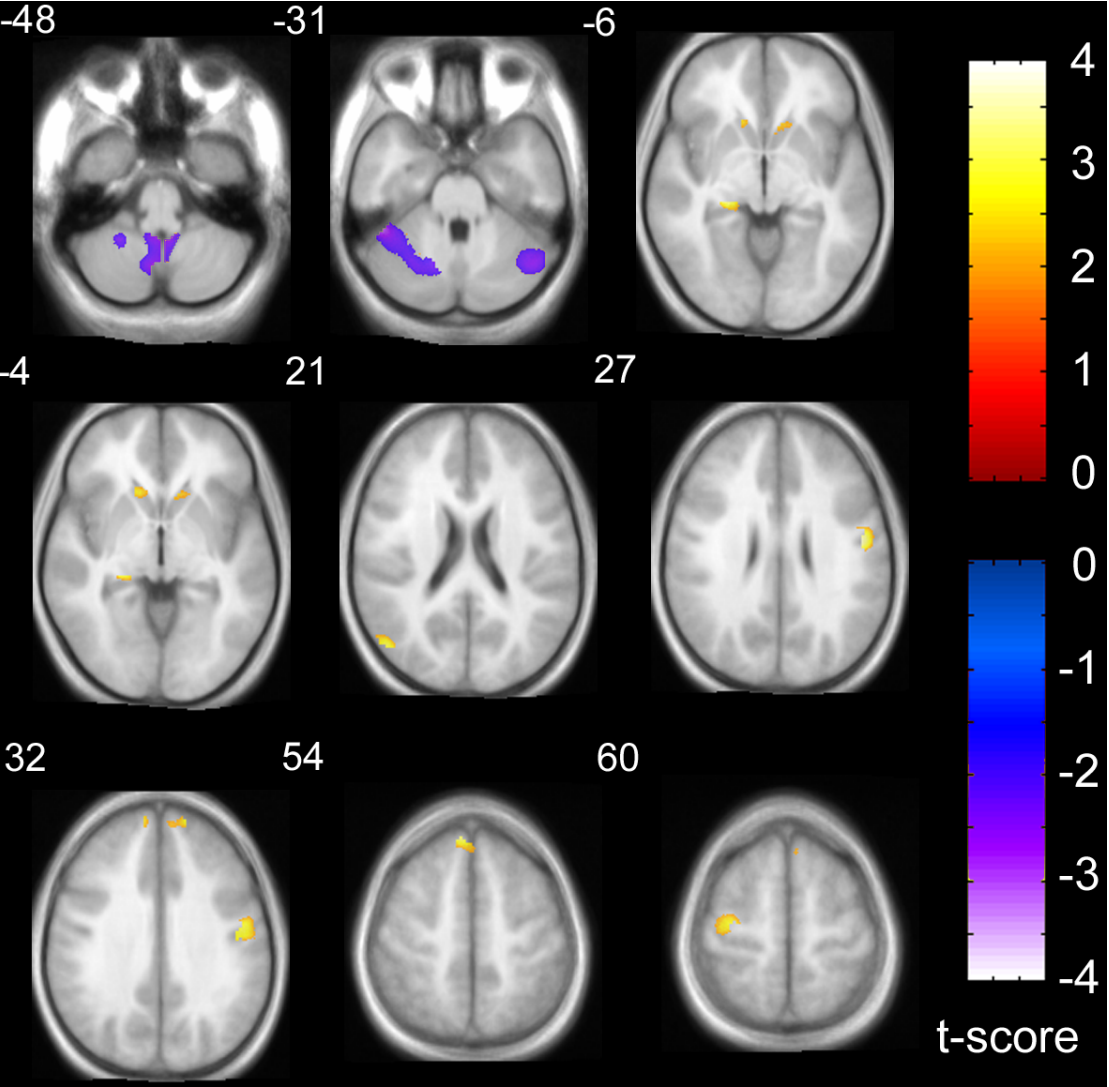
Gray matter volume differences between groups, overlaid onto average T1 image from study. Slice numbers (relative to AC-PC origin plane) are shown in upper left corner for each slice. Relative increases (autism > control) are shown in red-orange color scale corresponding to upper right color bar and relative decreases (control > autism) are shown in blue-violet color scale corresponding to lower right color bar. The display threshold is p < .01, uncorrected, but only clusters surviving small volume correction, p < .05, are illustrated (see Table 1 for coordinates and label descriptions).

Table 3 lists structures for which significant results were obtained after small volume correction in the ROIs for the study. Regional increases in GM volume in the autism group relative to control subjects, controlling for age and total GM, were observed in the left and right medial frontal gyri, left middle temporal and pre-central gyri, right fusiform gyrus, right post-central gyrus, left hippocampus, left and right caudate nuclei. Regional decreases in GM volume in the autism group were observed only in the cerebellum (Figure 2). No regions were identified outside of the a priori ROIs after correction for multiple comparisons at FDR < .05.

**Table 3:** Significant differences in GM volume between groups

**BRAIN REGION**	**T**	**CLUSTER SIZE**	**MNI COORDINATES**		
**Autism > Control**					
Frontal					
Left medial frontal gyrus	3.43	1459	-8	41	54
Right medial frontal gyrus	3.08	160	15	58	32
Left pre-central gyrus	3.11	693	-37	-15	60
Temporal					
Left middle temporal gyrus	3.24	1166	-50	-72	21
Right fusiform gyrus	3.09	354	38	-25	-28
Parietal					
Right post-central gyrus	3.76	1155	50	-9	27
Subcortical					
Left caudate nucleus	2.95	344	-13	21	-4
Right caudate nucleus	2.80	179	9	20	-3
Left hippocampus	3.48	274	-22	-28	-6
**Autism < Control**					
Cerebellum					
Left Cerebellar Crus I	3.33	11342	-46	-42	-31
Left Cerebellar Lobule VIII^†^	2.77	299	-25	-48	-48
Left Cerebellar Lobule IX*	3.36	11342	-5	-59	-59
Right Cerebellar Crus I	3.38	2792	53	-54	-36

Notes: Regions reported in table are significant after small volume correction, p < .05. ^† ^Multiple foci are reported within the same structure when local maxima are greater than 12 mm apart. All labels are derived from AAL atlas (see text). * this maximum is near midline and the cluster has significant overlap with the cerebellar vermis. It also is contiguous with the left cerebellar crus I finding, which is why the cluster size for the two is reported as the same number. The cluster size is the number of contiguous voxels in the cluster at an uncorrected p < .01.

### Demographic and symptom correlations

No significant correlations were observed between full scale IQ and regional GM volumes within the regions of interest in the study, even at p < .001 uncorrected for multiple comparisons. However, there were several regions that correlated significantly with age, although only the linear and quadratic components were significant (see Figure 1 and Table 2).

**Figure 3:**
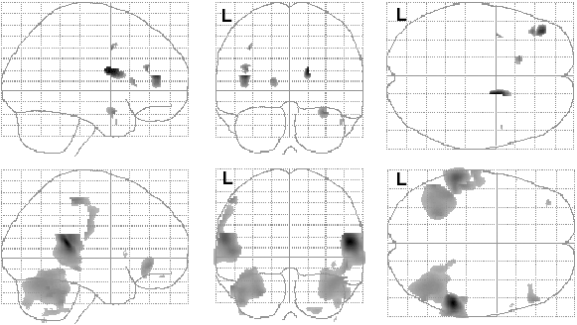
Correlations between regional gray matter volume and ADI-R Repetitive and Stereotyped Behavior Domain in the autism participants. Positive (top row) and negative (bottom row) partial correlations, corrected for total gray matter volume, are shown in glass brain projection. SPM(*t*) map display threshold is p < .01, uncorrected (Table 4 lists structures surviving small volume correction). L = left hemisphere.

**Figure 4:**
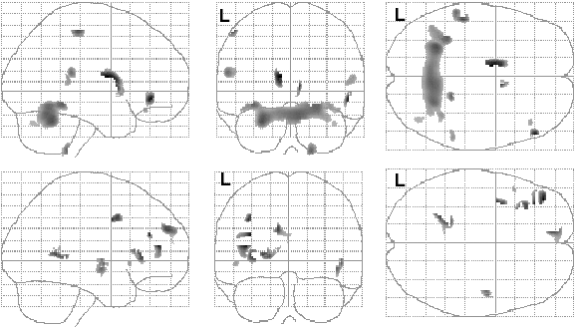
Correlations between regional gray matter volume and ADI-R Social and Communication Domain total scores in the autism participants. Positive (top row) and negative (bottom row) partial correlations, corrected for total gray matter volume, are shown in glass brain projection. SPM(t) map display threshold is p < .01, uncorrected (Table 4 lists structures surviving small volume correction). L = left hemisphere.

Table 4 lists regional partial correlations, controlling for total GM volume and correlations with other behavioral measures, for each of the symptom measures used in the study, after small volume correction. Regions with significant positive partial correlations with the ADI-R Repetitive and Stereotyped Behavior Domain, after small volume correction, include the left inferior frontal gyrus, caudate nuclei and right amygdala. Regions exhibiting negative correlations (i.e., smaller volumes associated with worse scores) included the superior temporal gyri, left post-central gyrus and cerebellar regions. For the ADI-R Social and Communication total score, positive correlations were present with the volumes of and caudate nucleus bilaterally, the left post-central gyrus, superior temporal gyri and cerebellar regions. Significant negative correlations were present with the left precuneus, left medial frontal and inferior frontal gyri, and left pre-central gyrus. The ADOS-G Severity measure exhibited significant negative correlations with the volumes of the left cerebellum (lobule IX) and right superior temporal gyrus. Figures 3 and 4 illustrate the ADI-R Repetitive and Stereotyped Behavior and Social/Communication Total symptom correlations with regional GM volume, respectively.

**Table 4:** Significant partial correlations between regional GM and symptom severity measures in the autism group.

**MEASURES**	**T**	**CLUSTER SIZE**	**MNI COORDINATES**		
**ADI-R Repetitive and Stereotyped Behaviors**					
Left Caudate Nucleus	2.99	145	-15	22	10
Right Caudate Nucleus	3.88	541	17	-1	19
Left Inferior Frontal Gyrus, Pars Triangularis	3.49	589	-42	42	14
Left Precentral Gyrus	3.23	40	-37	1	40
Right Amygdala	3.04	334	32	4	-21
Left Superior Temporal Gyrus	-5.07	7835	-60	-37	17
Right Superior Temporal Gyrus	-7.44	9192	55	-40	14
Left Postcentral Gyrus	-4.12	1476	-54	-56	-26
Left Inferior Frontal Gyrus	-3.16	42	-37	48	-17
Right Inferior Frontal Gyrus	-3.85	734	53	36	-7
Left Cerebellar Lobule VI	-4.04	13101	-54	-56	-26
Right Cerebellar Crus I	-3.72	12453	40	-63	-23
**ADI-R Social and Communication Total**					
Left Caudate Nucleus	3.88	613	-9	0	14
Right Caudate Nucleus	3.12	133	8	7	5
Left Post-central Gyrus	3.38	231	-52	-29	56
Left Superior Temporal Gyrus	2.79	186	57	-40	13
Right Superior Temporal Gyrus	3.07	466	-57	-36	19
Left Cerebellar Lobule VI*	3.33	13114	3	-60	-20
Right Cerebellar Lobule VI*	3.20	13114	8	-58	-21
Right Cerebellar Lobule VIII	3.15	249	19	-40	-55
Left Medial Frontal Gyrus	-3.29	518	-10	54	29
Left Inferior Frontal Gyrus, Pars Triangularis	-3.78	411	-40	44	14
Left Precentral Gyrus	-3.50	247	-37	1	38
Left Precuneus	-3.38	336	-24	-50	6
**ADOS-G Severity Measure**					
Left Cerebellar Lobule VI	-4.07	16420	-25	-52	-33
Right Superior Temporal Gyrus	-3.08	315	62	-40	15

Notes: The sign of the t-statistic indicates whether the correlation coefficients are positive or negative partial correlations. Regions reported in table are significant after small volume correction, p < .05. Cluster size is the number of contiguous voxels in cluster at p < .01 uncorrected. ^† ^All labels are derived from AAL atlas (see text). * This contiguous cluster has two local maxima, one on each side of the cerebellum.

## Discussion and conclusion

Most, if not all, of the regions in which we report differences between the autism and control groups have been previously associated with social cognitive processes in functional imaging and lesion studies. For example, functional MRI studies of Theory of Mind (TOM: i.e., the ability to make mental state inferences about others) have consistently reported activation of the medial aspect of the superior frontal gyrus, anterior to the region commonly referred to as the pre-supplementary motor cortex, despite using a wide variety of TOM tasks [72-76]. The precuneus region of the medial parietal lobe is also observed to be active in many TOM experiments, may be particularly important in self awareness and is heavily interconnected to the medial prefrontal regions and the dorsal striatum [77]. The posterior middle temporal gyrus/sulcus region has a role in biologically relevant motion perception, intentionality of eye gaze direction and inferring intentionality from stories, and is often active in functional imaging of TOM tasks [72,75,78-80]. Many of these same regions have been reported to be activated differently in people with autism and their first degree relatives while performing TOM tasks, relative to the activation seen in matched controls [72,81].

We found no significant correlations between full scale IQ and regional GM volume in our sample. Although this was a study of GM volume, rather than thickness, our findings our consistent with Hadjikhani et al. [57], who used the latter methodology in a recent study on autism. Taken together, these findings suggest that caution is warranted in using IQ as a covariate in anatomical studies of autism (e.g., see [18]). However, age was highly correlated with the volume of the hippocampus and surrounding region in our sample, which is consistent with the finding of Good et al. [67], who first described the optimized VBM approach in the literature and found positive correlations between age and medial temporal lobe volume in large sample of adults when controlling for total brain volume. In our sample, this might be driven by the lower end of the age range. However, only the linear component for age was significant. A study including a larger number of older adults might be able to demonstrate an asymptote on aging effects on the hippocampal region (i.e., a significant quadratic term).

The increased left hippocampal volume in the autism group, relative to the control group, is consistent with the findings reported in Rojas et al. [2]. This is expected given that almost 60 percent of the autism subjects from this study also participated in that paper, which used hand-tracing methods to assess the hippocampus. As discussed in the introduction, however, both increases and decreases in hippocampal volume have been reported in autism by hand tracing methods. To our knowledge, this is the first report of hippocampal volume change, increase or decrease, in autism using VBM methodology. A recent paper by Testa et al. [82] comparing hand-tracing to VBM for detecting hippocampal atrophy in Alzheimer's Disease provided data supporting the comparability of the two methods for hippocampal volumetry.

To our knowledge, this is the first VBM study in autism to utilize symptom measures from the ADI-R and ADOS-G to examine correlations with both cortical and subcortical GM. Hadjikhani et al. [57] performed such correlations, but only with regional cortical thickness, not with subcortical structural measures. Our significant positive correlation between caudate nucleus volume and the ADI-R Repetitive and Stereotyped Behavior domain is supportive of the same association reported in the manual tracing study of Hollander et al. [36]. Caudate enlargement has also been reported in Obsessive-Compulsive Disorder [OCD: 83]. Interestingly, caudate nucleus activity in OCD has been reported to be higher in both resting and symptom provocation conditions in functional neuroimaging studies [84,85], and treatment with either serotonin agonist medications or behavior therapy have been shown to reduce this over-activity [86,87].

An alternative, but not necessarily mutually exclusive, interpretation of the caudate nucleus correlation is that it simply reflects disorder severity generally. In support of this notion is that the caudate nucleus volume was significantly associated with both with the ADI-R Social and Communication total score and with the Repetitive and Stereotyped Behaviors score. Arguing against this interpretation is the lack of correlation observed in these same structures with the ADOS-G Severity measure. These other structures, along with the superior temporal gyrus and cerebellum, have been heavily implicated in social cognitive and motor abilities. Taken together, the GM findings and the symptom correlations suggest that the regional GM differences are highly relevant to the behavioral phenotype in autism.

Some caution is warranted with respect to the caudate nucleus findings, however. Three of our autism participants had a past history of neuroleptic medication treatment, use of which has been previously associated with caudate volume increases in schizophrenia [e.g., [88,89]]. However, all of three of these were taking atypical antipsychotic agents and discontinued use of the drugs at least 2 years prior to participation in the study. Studies have suggested that caudate volume increases to neuroleptic treatment are associated more strongly with typical neuroleptics and that volumes decrease rapidly after neuroleptic treatment is withdrawn [90,91]. It therefore seems unlikely that the caudate volume enlargement in our sample could be explained by treatment by neuroleptic medications.

There are several limitations to the results of this study to consider. Among them, the non-isotropic voxel dimensions of the MRI scans may have introduced partial volume effects that influenced the tissue segmentation step of the VBM procedure. Additionally, the large age range of participants could have introduced variability that reduced the effect sizes for some structures we hypothesized would differ between groups (e.g., the amygdala), but for which we did not find group differences. We attempted to control for age effects statistically, by treating the effects of age as covariates in the main analysis. This may not be the most optimal design, however, and further studies that more tightly control the age range of the samples are warranted. Third, segmentation algorithms such as the one employed here on T1 weighted MRI data generally do not discriminate well between deep cerebellar nuclei and the surrounding cerebellar white matter (most of the nuclei are classified as white matter). Fourth, the lack of inclusion of female participants, while advantageous in terms of sample homogeneity, limits the generalizability to males with autism. Finally, the IQ of the control participants (mean = 119), while not strictly a limitation, should be mentioned, because it is worth noting that previous population-based studies have indicated a higher mean IQ level in the Denver metropolitan region than in the national standardization sample for the Wechsler scales [92].

## Competing interests

The author(s) declare that they have no competing interests.

## Authors' contributions

DR conceived of the study, made the main contribution to the design and analysis, performed all VBM analyses and drafted the manuscript. EP, EW, MR and JT participated in the design of the study. SR made a substantial contribution to the selection and use of the clinical measures reported in the study.

## Pre-publication history

The pre-publication history for this paper can be accessed here:


